# Natural compound Oblongifolin C confers gemcitabine resistance in pancreatic cancer by downregulating Src/MAPK/ERK pathways

**DOI:** 10.1038/s41419-018-0574-1

**Published:** 2018-05-10

**Authors:** Yang Li, Zhichao Xi, Xiaoqiong Chen, Shuangfan Cai, Chen Liang, Zhen Wang, Yingyi Li, Hongsheng Tan, Yuanzhi Lao, Hongxi Xu

**Affiliations:** 1 School of Pharmacy, Shanghai University of Traditional Chinese Medicine, Shanghai, 201203 P. R. China; 2 Engineering Research Center of Shanghai Colleges for TCM New Drug Discovery, Shanghai, 201203 P. R. China; 3 Cancer Research Institute, Fudan University Shanghai Cancer Center, Department of Oncology, Shanghai Medical College, Fudan University, Shanghai, China

## Abstract

Gemcitabine (GEM)-induced drug resistance is the major reason for the failure of chemotherapy in pancreatic cancer (PC). In this study, we found that Oblongifolin C (OC) efficiently inhibited PC cell proliferation by inducing G0/G1 arrest and apoptosis. Also, our mechanism study demonstrated that OC re-sensitized the GEM-resistant PC cells through the ubiquitin-proteasome-dependent degradation of Src, and then downregulating the MAPK pathway. Knockdown of Src plus OC resulted in a greater inhibitory effect in GEM-resistant PC cells. In contrast, Src overexpression reversed OC-mediated chemosensitization, thereby implicating Src in the action of OC. Moreover, our *in vivo* study showed that OC suppressed the tumor growth via the downregulation of Src, and enhanced the chemosensitivity of GEM-resistant PC to GEM. Overall, our results have revealed that OC is applicable as a promising agent for overcoming GEM-resistant PC, especially with aberrant Src expression.

## Introduction

Pancreatic adenocarcinoma is the most lethal cancer and has a poor prognosis. Gemcitabine (GEM), a cytotoxic nucleoside analog, is the clinical standard chemotherapy for pancreatic cancer (PC). The development of GEM resistance leads to a low response to chemotherapy and remains a significant limitation to its use^1^. Thus, agents that reverse GEM resistance and improve the chemosensitivity of chemotherapy in PC are needed.

Src, a membrane-associated non-receptor tyrosine kinase, is commonly overexpressed in most late-stage tumor tissues, and is an indicator of poor clinical prognosis^2–5^. Thus, Src has been a drug development target, and a number of tyrosine kinase inhibitors are currently undergoing clinical evaluation as cancer therapies^6,7^. Dasatinib, a dual Abl/Src inhibitor, has been approved by the Food and Drug Administration for the treatment of chronic myelogenous leukemia^8^. Recently, a significant amount of data show that aberrant activation of Src contributes to chemotherapy drug resistance in different types of cancers^9–11^. Activated Src kinase is also correlated with colorectal carcinoma cell resistance, and Dasatinib, as an Src inhibitor, could inhibit this protein and restore the sensitivity of liver metastatic colorectal carcinoma to oxaliplatin^12^.

Natural compounds are the main resources of drug development. The natural polyphenolic compound gallic acid could re-sensitized EGFR tyrosine kinase inhibitors though the inhibition of Src-Stat3-mediated signaling^13^. In this study, we have confirmed that Oblongifolin C (OC), a natural product isolated from *Garcinia yunnanensis*, efficiently inhibits cell proliferation and enhances the sensitivity of GEM-resistant PC *in vitro* and *in vivo* through downregulation Src/MAPK/ERK pathways. Our findings suggest that OC is a new promising candidate to overcome GEM resistance in PC with the aberrant expression of Src.

## Results

### OC inhibits the proliferation of parental and GEM-resistant PC by inducing G0/G1 arrest and apoptosis

**Fig. 1:**
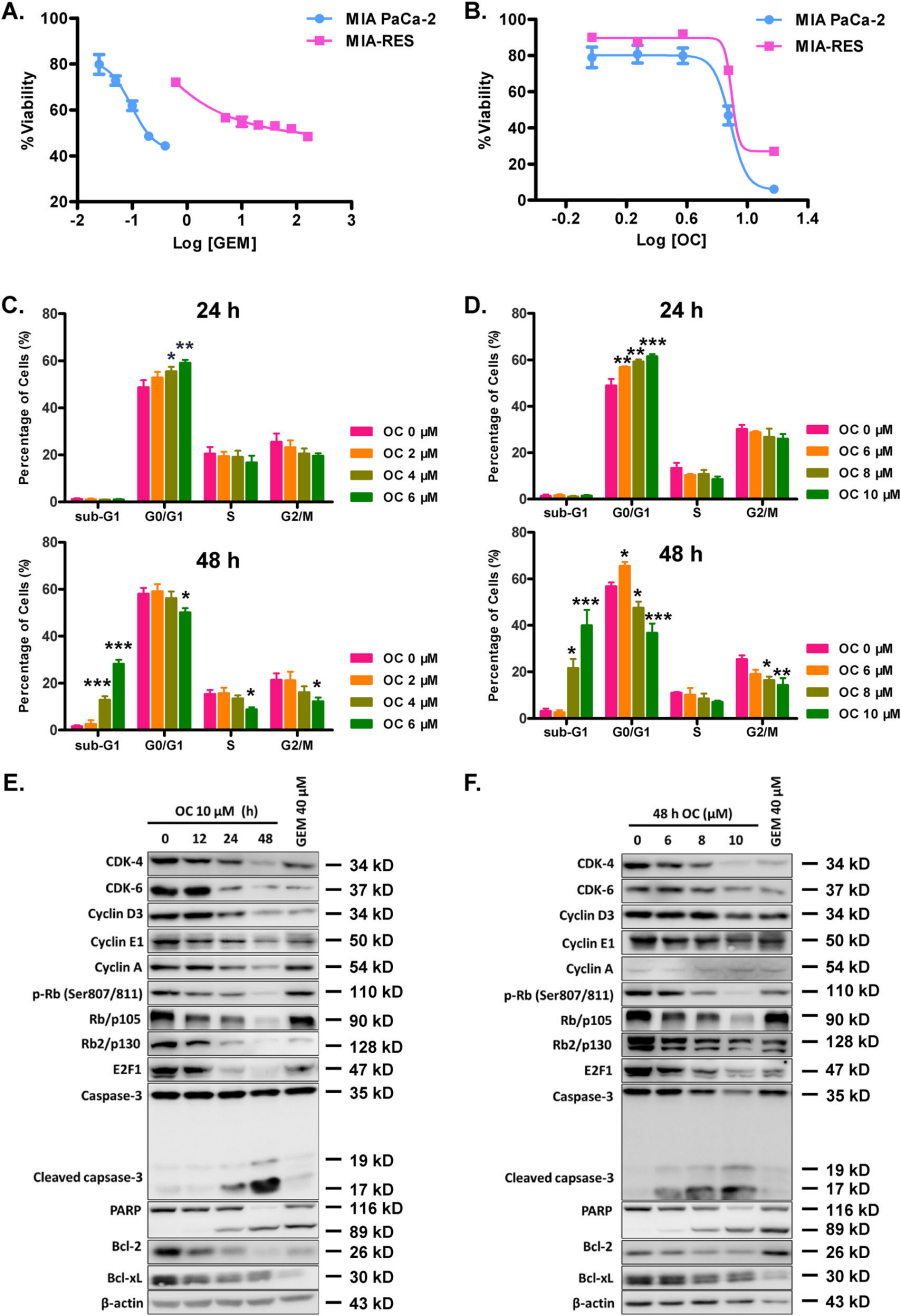
OC inhibits the proliferation of parental and GEM-resistant PC cells by inducing G0/G1 arrest and apoptosis. The cell viability of the parental human pancreatic cancer cell line MIA PaCa-2 and its GEM-resistant cell line MIA-RES were exposed to different concentrations of GEM **a** and OC **b** for 48 h, which were determined by a CCK8 assay. MIA PaCa-2 **c** and MIA-RES cells **d** were treated with indicated concentrations of OC for 24, 48 h, and subjected to flow cytometry analysis using propidium iodide staining. Data are expressed as the means ± SD of triplicate assays compared with the controls. *p < 0.05, **p < 0.01 and ***p < 0.001. MIA-RES cells were treated with OC for the indicated time periods **e** and concentrations **f**, and subjected to western blotting analysis using the indicated antibodies. *β*-actin was used as the protein loading control.

Our previous studies have been reported that OC exhibited multiple anticancer properties^14–16^. In this study, we first assessed the viability of five human PC cell lines, MIA PaCa-2, Capan-1, SW1990, PANC-1, and BxPC-3 upon OC treatment. As shown in Table 1, OC efficiently inhibited the proliferation of PC cells. Next, we induced MIA PaCa-2, Capan-1 into MIA-RES and Capan-1-RES via serially increasing the GEM concentrations, respectively. The IC_50_ values of GEM in the MIA-RES and Capan-1-RES cells increased markedly, which were 184 and more than 44 folds compared with their parental PC cells, respectively (Fig. 1a, Supplementary Fig. 1A and Table 2). Interestingly, Fig. 1b and supplementary 1B showed that OC still displayed cytotoxic effects against MIA-RES and Capan-1-RES cells with IC_50_ values of 9.86 ± 0.41 μM and 15.20 ± 0.35 μM, respectively, at 48 h. We then examined the cell cycle distribution and apoptosis using propidium iodide (PI) staining flow cytometric analysis. The results demonstrated that OC accumulated in the G0/G1 phase of MIA PaCa-2 (Fig. 1c and Supplementary Fig. 1C) and MIA-RES (Fig. 1d and Supplementary Fig. 1D) cells at 24 h in a dose-dependent manner, with G0/G1 cell numbers increasing significantly from 48.8% at 0 μM OC to 59.2% at 6 μM OC in MIA PaCa-2 and from 48.9% at 0 μM OC to 61.6% at 10 μM OC in MIA-RES, respectively. After treatment with OC for 48 h, a significant increase of Sub-G1 cells from 3.29% to 40.0% was observed in MIA-RES, and a similar effect with less potency was exerted in MIA PaCa-2 cells, with an increase from 1.62% to 28.2%. And the images of indicative cells were photographed by confocal microscopy (Supplementary Fig. 1E).

**Table 1:** IC_50_ values of OC in five different pancreatic cancer cell lines

Cell line	48 h	72 h
MIA PaCa-2	6.70 ± 0.55	5.68 ± 0.67
Capan-1	13.11 ± 0.22	7.89 ± 1.22
SW1990	22.15 ± 0.33	12.23 ± 0.64
PANC-1	13.90 ± 0.93	8.04 ± 0.41
BxPC-3	8.37 ± 2.08	6.97 ± 0.21

IC_50_ values of OC were determined with CCK8 assay in five different human pancreatic cancer cell lines with different concentrations for 48, 72 h, calculated by GraphPad Prism 5 software. IC_50_ values are shown as the mean ± SD of three independent experiments

**Table 2:** Comparison the sensitivity of OC and gemcitabine towards parental and GEM-resistant PC cells

Compound	MIA PaCa-2	MIA-RES	Capan-1	Capan-1-RES
OC	6.70 ± 0.55	9.86 ± 0.41	13.11 ± 0.22	15.20 ± 0.35
GEM	0.235 ± 0.012	43.38 ± 17.31	3.67 ± 0.12	> 160

IC_50_ values of OC and gemcitabine were determined with CCK8 assay in MIA PaCa-2, Capan-1 cells, and its GEM-resistant subline MIA-RES, Capan-1-RES cells for 48 h. IC_50_ values are shown as the mean ± SD of three independent experiments

We then were curious regarding how OC regulated cell cycle-related proteins. We found that OC treatment reduced the protein expression of cyclin D3, cyclin E1, and cyclin A, and CDKs 4 and 6 in a time- and dose-dependent manner (Fig. 1e and f) in MIA-RES cells, as well as the protein level of p-Rb (Ser807/811) and the tumor suppressor proteins with different members of Rb/p105 and Rb2/p130. In addition, OC decreased the protein level of E2F1, a transcription factor in both MIA-RES and MIA PaCa-2 cells (data not shown). Moreover, OC reduced the expression of Bcl-2 and Bcl-xL, two anti-apoptotic proteins, and activated the caspase-3 and PARP protein levels in PC cells.

### OC inhibits Src/MAPK/ERK signaling in MIA-RES cells

**Fig. 2:**
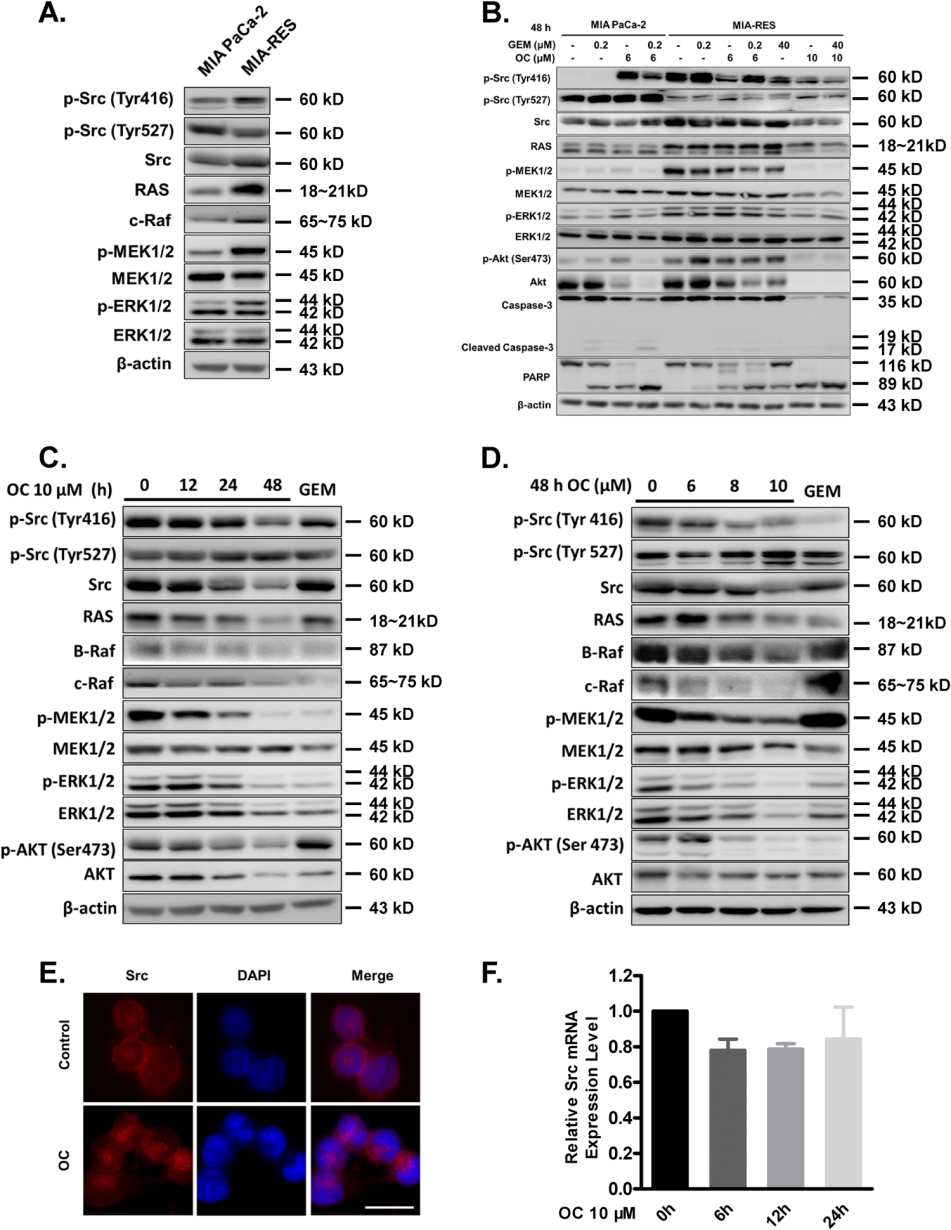
OC downregulates the Src/MAPK/ERK signaling in GEM-resistant pancreatic cancer cells. **a** Comparison the levels of Src/p-Src (Tyr416, Tyr527), Ras, MEK/p-MEK1/2, and ERK/p-ERK1/2 by immunoblotting analysis in MIA PaCa-2 and MIA-RES cells. **b** The indicated cells were treated with GEM (0.2 μM or 40 μM), with or without 6 μM or 10 μM OC for 48 h, then the cell lysates were analyzed by immunoblotting. *β*-actin was used as the protein loading control. **c** MIA-RES cells were treated with 10 μM OC for the indicated time periods (0–48 h) and indicated dose (0–10 μM) of OC for 48 h **d**, and treatment with 40 μM GEM for 48 h as control. The cell lysates were analyzed by immunoblotting. **e** Immunofluorescence staining of Src in MIA-RES cells treated with or without 4 μM OC for 12 h. **f** Relative mRNA levels of Src in MIA-RES cells were determined by qRT-PCR, treated with 10 μM of OC for the indicated time period (0–24 h).

It has been reported that the aberrant activation of Src contributes to chemotherapy drug resistance in different types of cancers^9–11^. To investigate whether the abnormal expression of Src involves in GEM-resistant PC cells, we compared the protein expressions of Src tyrosine kinases and its downstream mitogen-activated protein kinases (MAPKs) between MIA PaCa-2 and MIA-RES cells. Src activation is required the autophosphorylation site of Tyr416, and negatively regulated through phosphorylation of tyrosine 530 (avian Tyr527). Figure 2a shows that Src activity is highly expressed and constitutively activated in MIA-RES cells with the overexpression of Src tyrosine 416 phosphorylation and the faint band of phosphorylated Tyr527, compared with the parental MIA PaCa-2 cells. Consistently, its downstream MAPK signaling pathway was also activated. Next, we examine the consequences of Src/MAPK signaling after OC treatment in both cell lines. As shown in Fig. 2b, the Src/MAPK/ERK phosphorylation levels and AKT activity were remarkably decreased in MIA-RES cells after treatment with 10 μM OC, with or without 40 μM GEM for 48 h. Although treatment with lower doses of OC and GEM were less effective. In addition, OC could not modulate the parental MIA PaCa-2 cells through downregulation Src/MAPK pathways. In addition, Figs. 2c and 2d showed that OC inactivated the Src protein by phosphorylation of the negative regulatory tyrosine 527 and downregulation the expression of p-Src Tyr416. Meantime, OC inhibited the MAPKs and AKT activity in a time- and dose-dependent manner. These findings suggested that OC re-sensitized MIA-RES cells to GEM by downregulating Src/MAPK and AKT pathways. To elucidate the possible mechanism of OC against Src signal, immunofluorescence staining was applied to observe the translocation of Src in MIA-RES cells. As shown in Fig. 2e, Src was dissociated from the membrane into the cytoplasm upon of 4 μM OC treatment for 12 h, suggesting that OC might act on the Src localization and was followed with the inhibition of Src. In addition, the transcription of Src was determined by qRT-PCR and Fig. 2f, which showed that the mRNA levels of Src did not decrease following OC treatment, suggesting that OC does not directly influence the transcription of Src.

### Knockdown of Src plus OC enhances the chemosensitivity to GEM in GEM-resistant human PC cells

**Fig. 3:**
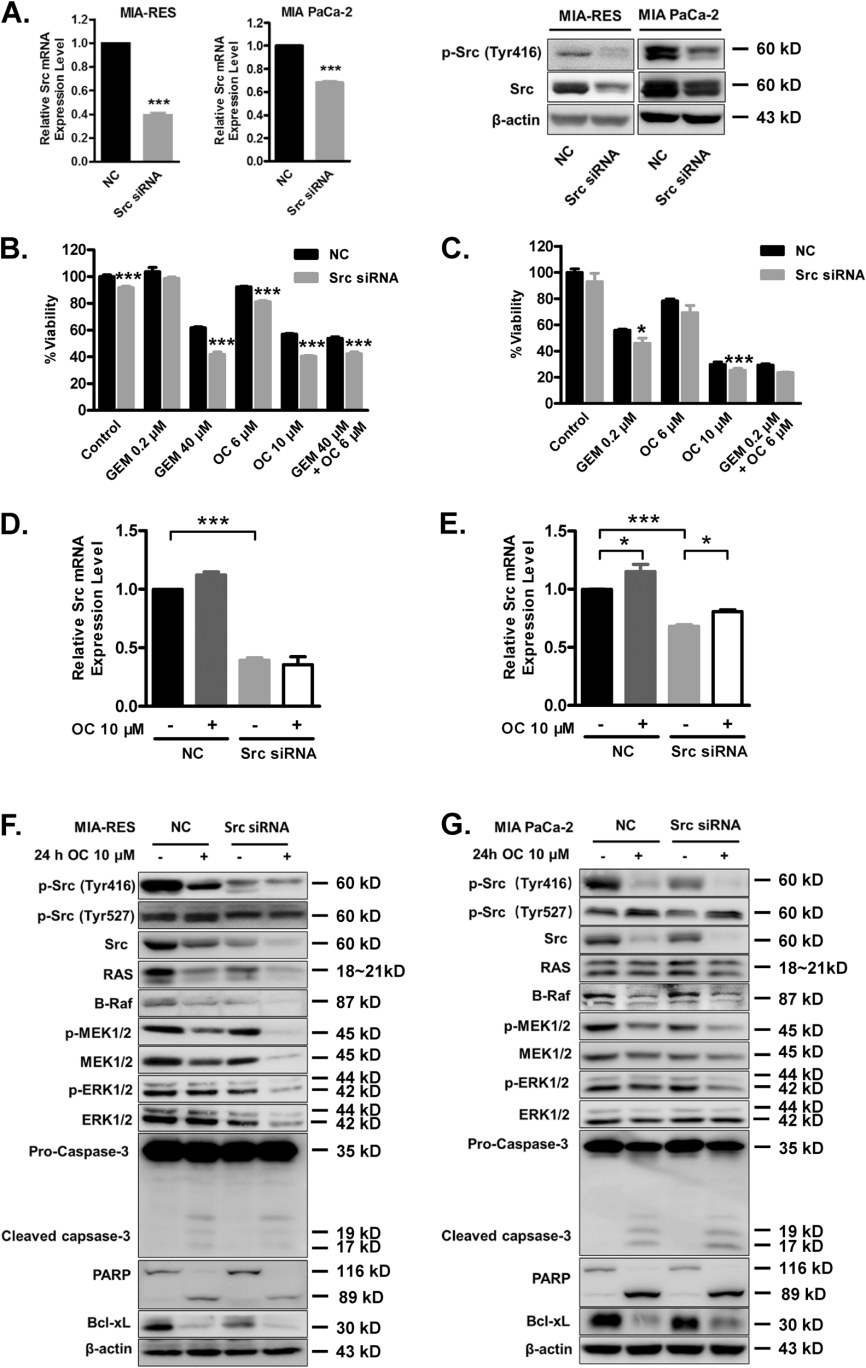
Knockdown of Src plus OC increases chemosensitivity of MIA-RES cells. **a** MIA-RES and MIA PaCa-2 cells were transfected with non-targeting siRNA (NC) or Src-targeting siRNA (Src siRNA) for 48 h. The expression of Src mRNA (left) and protein (right) were detected by qPCR and western blot analysis. TBP served as an endogenous control. *β*-actin was used as a loading control. Cell viability levels of MIA-RES **b** and MIA PaCa-2 cells **c** were measured by CCK8 assay in Src-silenced (Gray bars) and non-silenced cells (Black bars). Treatments are indicated at the bottom of the graph for 48 h. Data represent the average of three independent experiments ± standard deviation. *p < 0.05 and **p < 0.01 ***p < 0.001. Relative Src mRNA expression levels of MIA-RES **d** and MIA PaCa-2 cells **e** were determined in Src-silenced and non-silenced cells, treated with or without 10 μM OC for 24 h. MIA-RES **f** and MIA PaCa-2 cells **g** were transfected with non-targeting siRNA (NC) or Src-targeting siRNA for 48 h, followed by DMSO or OC treatment for 24 h. Cell lysates were analyzed by immunoblotting. *β*-actin expression was used as a loading control.

Considering the high expression levels of Src and its downstream MAPKs in GEM-resistant PC cells, we hypothesized that abnormal expression of Src was responsible for chemoresistance. To verify this hypothesis, Src-specific siRNA was used to knockdown Src in GEM-resistant PC cells and its parental cells. As shown in Fig. 3a and supplementary Figure 2A, a pool of c-Src siRNAs, which include 4 target-specific 19–25 nt siRNAs, exhibited strong Src knockdown efficacies in both the mRNA expression and protein levels. Src activity was severely suppressed, with the autophosphorylated tyrosine 416 site downregulated in both Src-silencing cells. As predicated, the knockdown of the endogenous expression of Src significantly mediated the sensitization to cell death in MIA-RES cells when treated with OC or GEM (Fig. 3b). In Src knockdown cells, 40 μM GEM treatment decreased the MIA-RES cell viability to 41.8%, which is a nearly 20% reduction compared to the non-silenced cells (61.8%). Interestingly, treatment with 10 μM OC in Src knockdown cells showed almost the same effects as 40 μM GEM, the cell viability decreased from 66.5% in mock cells to 49.4% when treated with 10 μM OC, whereas, co-treatment with 6 μM of OC and 40 μM GEM showed slight synergetic effects. Compared with MIA-RES cells, the parental MIA PaCa-2 cells showed a slightly inhibitory effect when treated with OC in Src-silencing cells (Fig. 3c). And similar results were obtained in Capan-1-RES and Capan-1 cells, which are shown in Supplementary Figure 2C and 2D. To understand the mechanism underlying the enhanced antitumor effect of OC following the Src knockdown, we first assessed the mRNA level of Src. As shown in Figs. 3d and 3e, Src mRNA was slightly affected by OC treatment before and after silencing Src expression. Second, we also examined the activities of MAPKs in the indicated cells before and after OC treatment. It is due to that Src is associated with the promotion of MAPK-dependent cell proliferation and apoptosis^17,18^. Knockdown of the expression of Src significantly attenuated the activity of Src, with the decrease level of its autophosphorylated tyrosine 416 site, and also the expression of its downstream in MIA-RES cells. In addition, treatment of OC after silencing Src expression resulted in a greater inhibitory effect, with the expression of Src/MEK/ERK barely detectable (Fig. 3f). While comparing the protein expression of negative control and its Src-silenced MIA PaCa-2 cells, OC treatment had a minimal effect on the downstream protein of Src, which is insufficient to sensitize cells to death (Fig. 3g). Knockdown of Src had little effect on parental MIA PaCa-2 cells, one possibility is that the low basal expression of Src, and Src/MAPK pathways is not the major survival signaling.

Taken together, our data indicated that OC efficiently sensitized GEM-resistant PC cell death via downregulating Src expression, and it exhibited better effects after depleting Src expression in PC cells.

### Overexpression of Src reverts the effect of OC and enhances the chemoresistant of PC cells

**Fig. 4:**
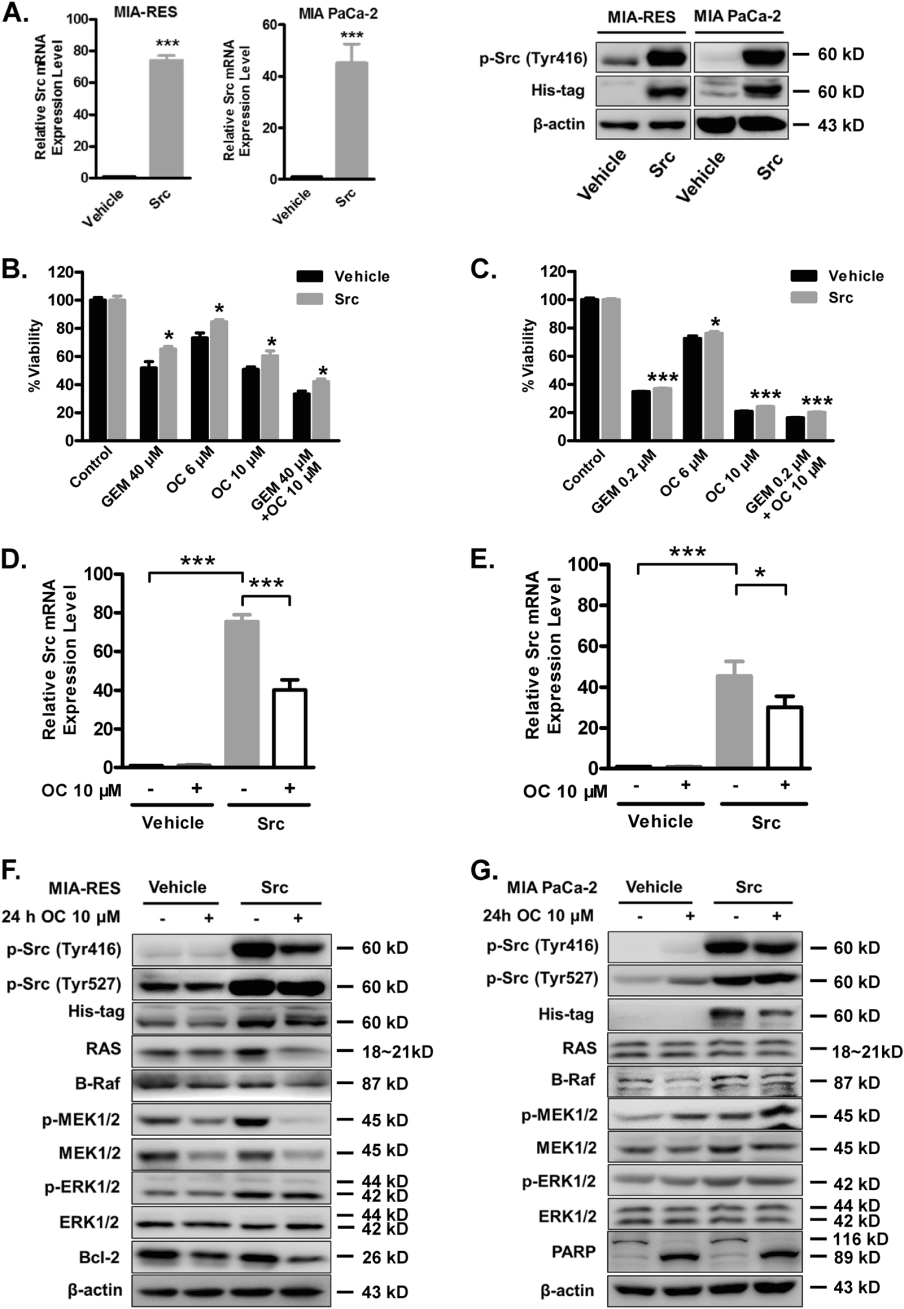
Overexpression of Src reverts the effect of OC and enhances the chemoresistant of pancreatic cancer cells. **a** MIA-RES and MIA PaCa-2 cells were transfected with 2 μg Src plasmid or empty vector (Vehicle) for 24 h. The expression of Src mRNA (left) and protein (right) were detected by qPCR and western blot analysis. TBP served as an endogenous control. *β*-actin was used as a loading control. Cell viability levels of MIA-RES **b** and MIA PaCa-2 **c** were measured by CCK8 assay in Src overexpression (Gray bars) and empty vector (Black bars). Treatments are indicated at the bottom of the graph for 48 h. Data represent the average of three independent experiments ± standard deviation. *p < 0.05 and **p < 0.01 ***p < 0.001. Relative Src mRNA expression levels of MIA-RES **d** and MIA PaCa-2 cells **e** were determined after transfected with empty or Src plasmid, followed by the treatment with or without 10 μM OC for 24 h. **f** MIA-RES and MIA PaCa-2 cells **g** were transfected with Src expression vector or empty vector for 24 h, followed by DMSO or OC treatment for 24 h. Cell lysates were analyzed by immunoblotting. *β*-actin expression was used as a loading control.

To further verify the effect of OC on Src kinase, we overexpressed Src to examine whether the effect of OC still existed. The transfection efficiencies of Src were shown in Fig. 4a and supplementary Figure 2B. Transfection of Src plasmid obviously increased the levels of mRNA expression and protein expression in PC cells. As showed in Figs. 4b, 4c and Supplementary 2E, 2 F, neither the Src plasmid nor the empty expression vector alone significantly altered the observed cellular viability. Whereas, overexpression of Src increased chemoresistant to GEM both in parental and GEM-resistant PC cells. To a certain extent, Src overexpression also reverted the effect of OC, or their combination. These results suggested that Src overexpression led to a low response of GEM in PC and reversed OC-mediated chemosensitization. In addition, OC not only downregulate the ectopic protein expression of Src but also the mRNA level when transfection with exogenous Src plasmids into the two indicated cell lines (Figs. 4d and 4e). Figure 4f shows that Src overexpression activated MAPKs, and OC could partially block Src activity and MAPK signaling pathways, whereas OC exhibited little effect on MIA PaCa-2 cells (Fig. 4g). These findings suggest that OC-induced downregulation of Src have a critical role in inducing cell death of GEM-resistant PC cells.

### OC promotes Src protein degradation by the ubiquitin-proteasome pathway

**Fig. 5:**
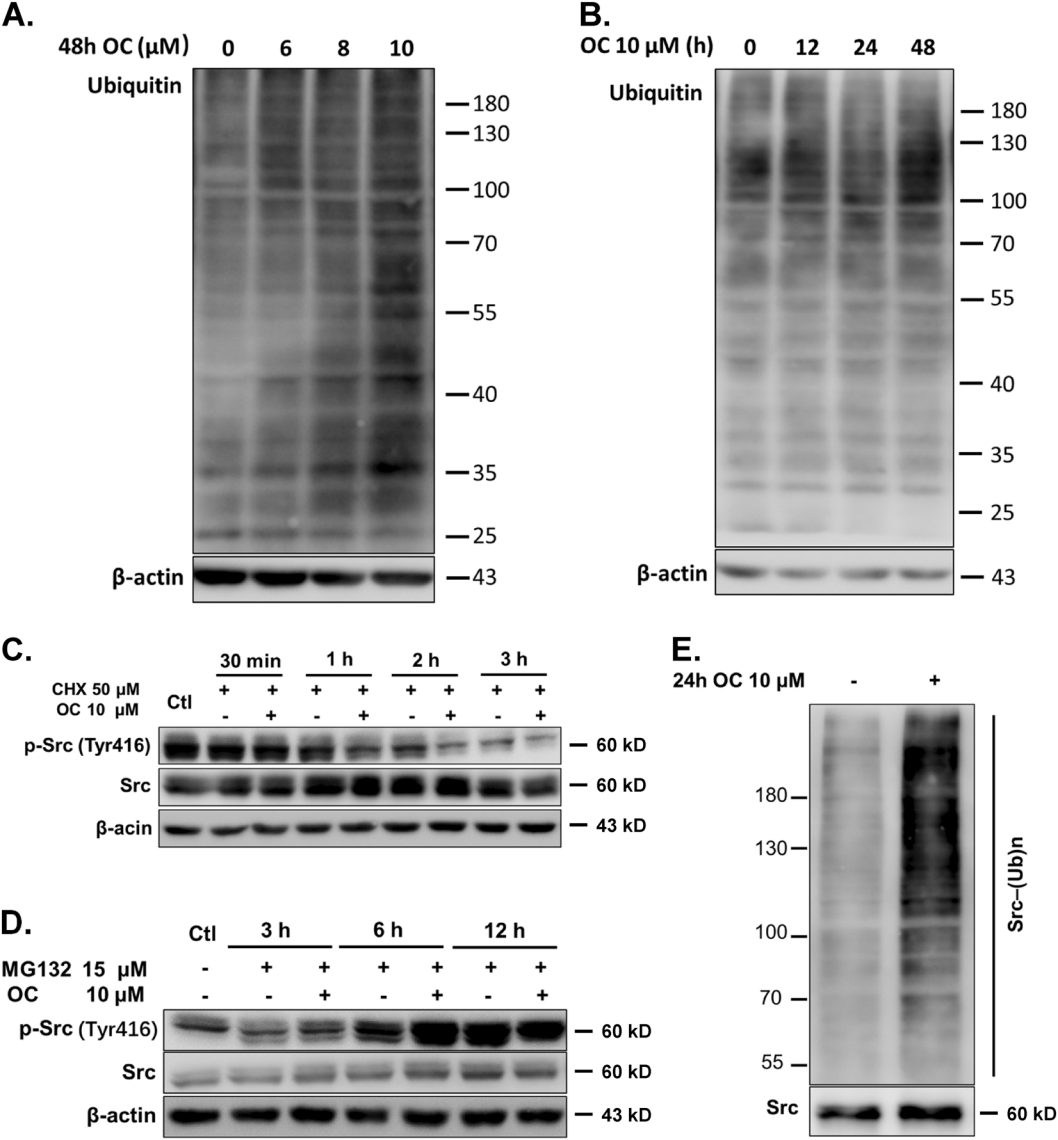
OC promotes Src protein degradation by the ubiquitin-proteasome pathway. MIA-RES cells were treated with OC for the indicated dose (0–10 μM) **a** and indicated time periods (0–48 h) **b**, then the level of ubiquitination was detected by western blotting using an anti-ubiquitin antibody. **c** MIA-RES cells were treated with 50 μM cycloheximide (CHX) with or without 10 μM OC for the indicated time and the cell lysates were analyzed by immunoblotting. *β*-actin served as a loading control. **d** MIA-RES cells were co-treated with 15 μM MG132 with or without 10 μM OC and harvested at 0, 3, 6,12, and 24 h after treatment for western blotting analysis. *β*-actin served as a loading control. **e** MIA-RES cells were transfected with 2 μg Src plasmid for 24 h, and treated with 10 μM OC for 24 h. The cells were treated with 15 μM MG132 for 6 h before collected, and 6xHis-tagged proteins of Src were purified with Ni-NTA Agarose beads, followed by western blotting analyses to detect the level of Src ubiquitination.

As OC sensitized the MIA-RES cells through the downregulation of Src expression, we further explored the mechanism that how OC regulated Src in PC cells. First, we examined the overall ubiquitination status upon OC treatment. Figures 5a and 5b showed that the ubiquitination level was increased upon OC treatment in a dose- and time-dependent manner. Several studies demonstrated that the active form of Src is required in a process of ubiquitin-proteasome-dependent degradation of Src^19,20^. Then, we exposed MIA-RES cells to the synthesis inhibitor by cycloheximide to investigate Src stability. The combined treatment of cycloheximide (CHX) with OC dramatically reduced protein stability of Src, with the active form of autophosphorylation tyrosine 416 site in predominant (Fig. 5c). Furthermore, we used MG132 to inhibit 26 s proteasome activity in MIA-RES cells. We found that the Src expression was enriched by OC treatment for 6 h, suggesting that Src protein is degraded by the ubiquitin-proteasome pathway (Fig. 5d). We then performed immunoprecipitation to examine the directly effect of OC on Src ubiquitination. We overexpressed his-tagged Src in HEK293T cells and observed the ubiquitination level of Src upon OC treatment. As shown in Fig. 5e, OC increased the ubiquitination level of Src at 24 h.

### OC suppresses orthotopic pancreatic tumor growth and improves the chemosensitivity of GEM *in vivo*

**Fig. 6:**
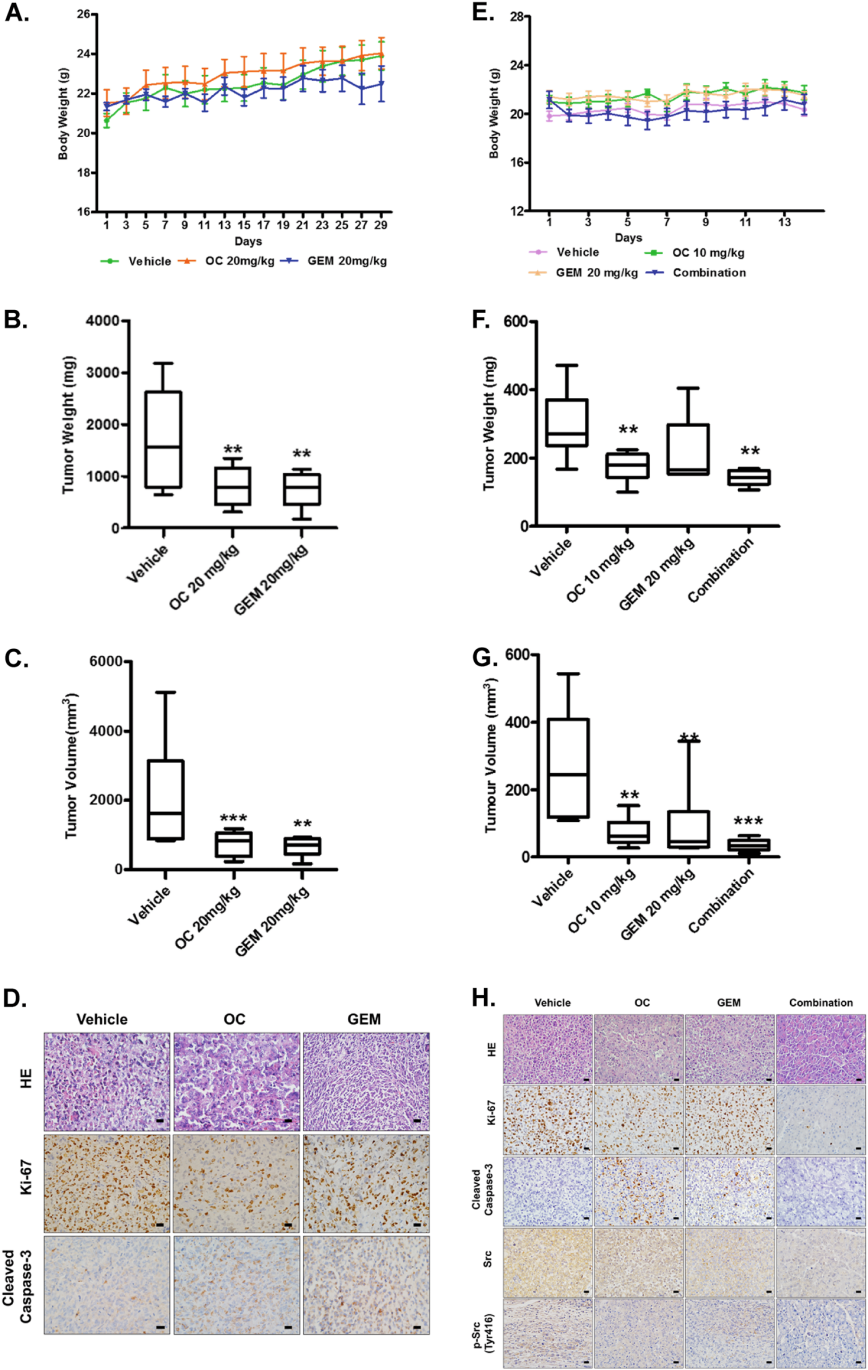
OC suppresses orthotopic pancreatic tumor growth and improves the chemosensitivity of GEM *in vivo*. MIA PaCa-2 cells (1 × 10^6^ cells per mouse) were implanted directly into the pancreas of nude mice. One week later, the mice received intraperitoneal injections of either vehicle control (0.05% DMSO in normal saline), 20 mg/kg OC every 2 days, or GEM 20 mg/kg twice a week. Body weights **a** of the animals were measured every other day. After four weeks, the mice were killed, tumor weight **b**, and tumor volume **c** were measured. *p < 0.05, **p < 0.01, ***p < 0.001 versus the corresponding control groups (*n* = 6). **d** Paraffin-embedded orthotopic tumor tissues sections were stained with HE or Ki-67, cleaved caspase-3 antibodies (Scale bar: 20 *μ*m). For the GEM-resistant cellular orthotopic implantation, 1 × 10^6^ MIA-RES cells were injected into the pancreas of each mice. One week later, the mice received intraperitoneal injections of either vehicle control (0.05% DMSO in normal saline), OC (10 mg/kg) every day and GEM (20 mg/kg) twice a week alone or in combination with OC. Body weights **e** of the animals were measured every day. After 2 weeks of treatment, the mice were killed, tumor weight **f**, and tumor volume **g** were measured. **h** Paraffin-embedded orthotopic tumor tissues sections were stained with HE, Ki-67, Src and p-Src (Tyr416), cleaved caspase-3 antibodies (Scale bar: 20 *μ*m).

To verify the inhibitory effect of OC in parental and GEM-resistant PC *in vivo*, we established two orthotopic mouse models using MIA PACA-2 and MIA-RES cells, respectively. No significant differences in body weights between the vehicle and OC treatment groups were observed, whereas the mice in GEM group were slightly decreased after day 23 (Fig. 6a). In the orthotopic animal models of parental PC cells, intraperitoneal administration of 20 mg/kg OC twice a day showed a similar effect as GEM, with a remarkable reduction of the xenograft tumor weight and tumor volume compared with the control mice (Figs. 6b, 6c and Supplementary Fig. 3A). In addition, immunohistochemical analysis revealed that OC reduced the number of proliferating Ki-67-positive cells and induced cleaved caspase-3 (Fig. 6d and Supplementary Fig. 3C).

To verify whether OC could enhance the chemosensitivity of GEM in GEM-resistant PC *in vivo*, orthotopic MIA-RES xenograft-bearing mice were divided into four groups: control, OC, GEM, and OC + GEM. Mice treated with GEM exhibited a tendency toward resistance effects, while OC + GEM exhibited better inhibitory effects on tumor growth than single drug treatment (Figs. 6f, 6g and Supplementary Fig. 3B) with no obvious changes of body weight of the animals (Fig. 6e). Consistent with the immunohistochemical staining of Ki-67 and cleaved caspase-3 (Fig. 6h and Supplementary Fig. 3D), OC activated apoptosis in OC treatment tumor tissue, and the lowest cell proliferation rate (Ki-67-positive cells) were appeared in the combination group. Also, we confirmed that OC treatment result in a similarly downregulation of Src expression *in vivo*, with the Src-positive cells in the OC treatment group decreased from 62.6% to 46.9%, and a significant reduce (4.9%) in combination group (*p* < 0.001).

Taken together, our results suggest that OC exert inhibitory effects on pancreatic tumor growth *in vivo* and improve the sensitivity of GEM through downregulating Src expression.

## Discussion

Several publications mentioned that the natural products isolated from *Garcinia* species have been used for chemosensitizers in different types of cancer. α-Mangostin, a natural xanthone derived from *Garcinia mangostana L*, selectively inhibited ABCG2-mediated drug transport and reversed MDR in ABCG2-overexpressing MDR cancer cells^21^. Li F *et al*. showed that Garcinol, a natural polyisoprenylated benzophenone derivative (PPAP), chemosensitized head and neck squamous cell carcinoma b negatively regulating constitutive cisplatin-induced nuclear factor-kB activation and the expression of oncogenic gene product^22^.

Src, a membrane-associated non-receptor tyrosine kinase, is constitutively active in pancreatic carcinoma tissue^23^. More than 70% of oncogenes code for tyrosine kinases, so the inactivation of tyrosine kinases is a rational approach for PC therapy^24,25^. Previous studies also reported that Src kinase overactivity represents a chemoresistance mechanism and Src inhibition reverts chemoresistance in many cancer cells^26–28^. For these reasons, our study aims to focus on the Src inhibitor not only as an antitumor agent but also the potential ability to overcome the chemoresistance of acquired resistance PC.

In our study, we first demonstrated that OC, a natural PPAP, exhibited a reversal effect on GEM-resistant PC through downregulating Src protein via ubiquitin-proteasome pathway. While the transition of Src inhibitors to the clinic was limited by its toxicity, which was also mentioned in Johnson’s publication^29^. In contrast, our study showed great growth inhibitory effects of OC in GEM-resistant PC without obvious side effects in nude mice. These results indicated that OC is a potential Src inhibitor in GEM-resistant PC.

In summary, our present study elucidated for the first time that OC represented an alternative therapy to overcome GEM-resistant PC growth by interfering with cellular tyrosine kinase Src both *in vitro* and *in vivo*.

## Material and Methods

### Cell cultures and development of GEM-resistant PC cells

Human pancreatic carcinoma cell lines MIA PaCa-2, PANC-1, and BxPC-3, Capan-1, SW1990 were purchased from the American Type Culture Collection. All the cell culture protocols and the development of MIA PaCa-2, Capan-1 into GEM-resistant MIA-RES and Capan-1-RES cells were followed as described previously^30^.

### Cell viability assay

The cells were exposed to OC or GEM treatment for 48 h 1 day after seeding in 96-well plates. Cell viability was measured using the Cell Counting Kit (CCK8) (Yeasen, China), and the absorbance was measured at 470 nm using a microplate reader (FLUOstar Omega, BMG Labtech, Germany). The IC_50_ values were calculated from dose–response curves using GraphPad Prism 5 (La Jolla, California, USA).

### Propidium iodide staining for flow cytometry

MIA PaCa-2 (3 × 10^5^) and MIA-RES (4 × 10^5^) cells were plated in a 60-mm^2^ culture plate and maintained with OC with associated concentrations. After 24 and 48 h, the cells were collected and fixed with 70% ethanol in phosphate-buffered saline (PBS) at 4 °C. Then, the cells were resuspended with PBS, containing 100 μg/mL RNase (Sigma, R6513) and 20 µg/mL PI (Sigma, P4170), and incubated for 1 h at 37 °C. Finally, the cells were determined using a flow cytometer (BD Biosciences, San Jose, CA) and analyzed using FlowJo software (version VX).

### Western blotting assay

The detailed protocol has been described previously^31^. The information of antibodies was listed in Supplementary Table 1.

### Transfection

c-Src siRNA (sc-29228; Santa Cruz, CA, USA) and negative control siRNA (sc-37007) were transfected with Lipofectamine RNAiMAX Reagents (Invitrogen) according to the manufacturer’s instructions. The oligonucleotide sequences were listed in Supplementary Table 2.

The Src plasmid was cloned into the *Xho*I and *Kpn*I restriction site of the GV219 vector (Genechem, Shanghai, China) and 2 μg of plasmid was transfected for 24 h using Lipofectamine 2000 (Life technologies), as instructed by the manufacturer.

### RNA extraction and quantitative real-time PCR

qRT-PCR was performed as described previously^32^. The PCR primers were listed in Supplementary Table 3. All the results were normalized to TATA box-binding protein (TBP).

### Immunofluorescent staining

MIA-RES cells were allowed to adhere overnight and exposure to 4 μM OC for 12 h. Then, the cells were fixed with 4% paraformaldehyde/PBS, blocked with 10% BSA in PBS and incubated with anti-Src antibodies (1:50; CST#2109), followed by Cy3-labeled secondary antibody (Beyotime, Shanghai, China). Images were visualized using an inverted fluorescent microscope (Olympus, Japan).

### Measurement of Src protein stability and ubiquitination

MIA-RES cells were co-treated with 10 μM OC with 50 μM CHX. After treatment for a given time, the cells were harvested for the western blot analysis of the Src protein level. For analysis of Src ubiquitination, HEK 293 T cells were transfected with his-tagged Src vectors. One day later, the cells were treated with 10 µM OC for 24 h, and treated with 15 µM MG123 for 6 h before harvest. His-tagged Src was purified with Ni-NTA Agarose beads according to the manufacturers’ protocols (Qiagen). The ubiquitination levels and the amount of Src-his-tagged protein in the immunoprecipitation product were detected by western blotting.

### Orthotopic tumor implantation in nude mice

Five-week-old female BALB/c nude mice were purchased from the Experimental Animal Center of the Chinese Academy of Science (Shanghai, China) and kept in a pathogen-free environment. All animal experiments were performed in accordance with the national guideline as described previously^33^. For the parental GEM-sensitive cellular orthotopic implantation, 1 × 10^6^ MIA PaCa-2 cells were injected into the pancreatic tail of each mouse. The mice were randomly divided into three groups (6 mice per group) for intraperitoneal treatment for 4 weeks with either vehicle control (0.5% dimethyl sulfoxide (DMSO), 0.5% Tween 80) (Group 1); OC, 20 mg/kg every 2 days (Group 2); or GEM, 20 mg/kg twice a week (Group 3).

For the GEM-resistant cellular orthotopic implantation, 1 × 10^6^ MIA-RES cells were injected into the pancreas of each mice. Twenty-four mice were divided into four groups as follows for intraperitoneal treatment: vehicle control (0.5% DMSO, 0.5% Tween 80) (Group 1); OC, 10 mg/kg every day (Group 2); GEM, 20 mg/kg twice a week (Group 3), combination OC 10 mg/kg every day and GEM 20 mg/kg twice a week (Group 4), begun 1 week after tumor implantation (day 1).

A few weeks later, the mice were sacrificed, and the tumor tissues were removed and subjected to immunohistochemical analysis. Tumor volumes were calculated using the following formula: (length × width × height) × *π* / 6.

### Immunohistochemistry

Tumors were fixed in 10% neutral-buffered paraformaldehyde. Next, the samples were embedded in paraffin, stained with hematoxylin and eosin, cleaved caspase-3 (ab9664), Src (CST, 2109), p-Src Y418 (ab4816), and Ki-67 (EPITMICS, 2642-1). Finally, the sections were mounted with DPX Mountant (Sigma, 317616) for histological analysis.

### Statistical analysis

The statistical software SPSS version 15.0 was used for the statistical analysis. Student’s *t*-test was used for comparison between the two different groups, and ANOVA analysis was used for the multiple comparisons. All *p* values < 0.05 were considered statistically significant. Statistical significance was indicated (*p < 0.05; **p < 0.01; ***p < 0.001), and all tests were two-tailed.

## Electronic supplementary material

Supplementary Table 1

Supplementary Table 2

Supplementary Table 3

Supplementary Figure 1

Supplementary Figure 2

Supplementary Figure 3

Supplementary figure legends
