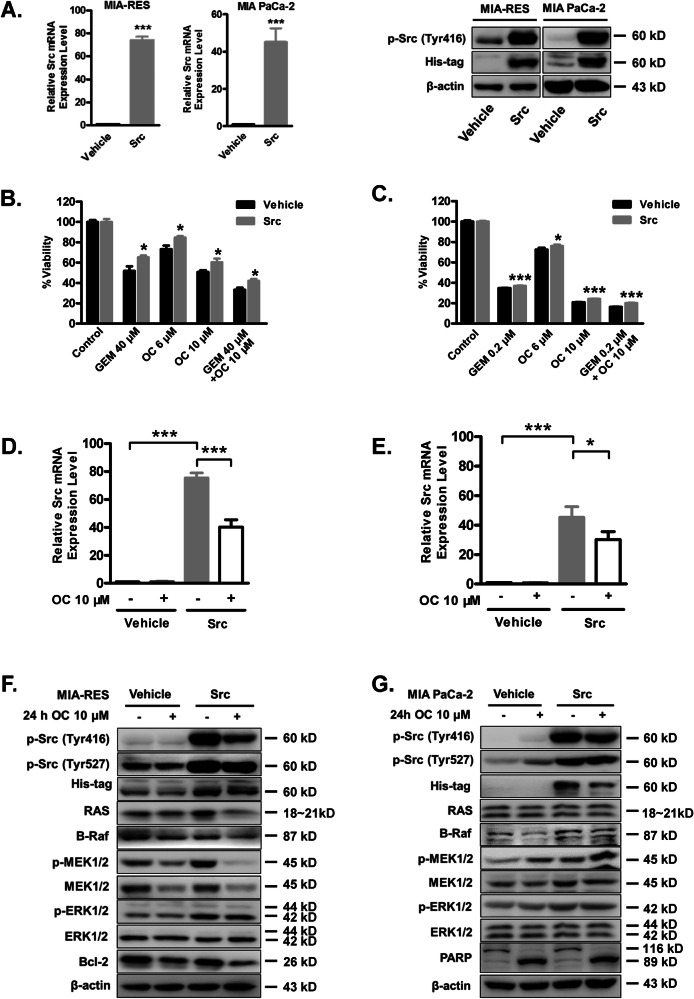# Correction to: Natural compound Oblongifolin C confers gemcitabine resistance in pancreatic cancer by downregulating Src/MAPK/ERK pathways

**DOI:** 10.1038/s41419-025-07495-2

**Published:** 2025-04-25

**Authors:** Yang Li, Zhichao Xi, Xiaoqiong Chen, Shuangfan Cai, Chen Liang, Zhen Wang, Yingyi Li, Hongsheng Tan, Yuanzhi Lao, Hongxi Xu

**Affiliations:** 1 School of Pharmacy, Shanghai University of Traditional Chinese Medicine, Shanghai, 201203 P. R. China; 2 Engineering Research Center of Shanghai Colleges for TCM New Drug Discovery, Shanghai, 201203 P. R. China; 3 Cancer Research Institute, Fudan University Shanghai Cancer Center, Department of Oncology, Shanghai Medical College, Fudan University, Shanghai, China

Correction to: *Cell Death and Disease* 10.1038/s41419-018-0574-1, published online 10 May 2018

Upon a thorough review of the published content, we recently identified mistakes in Fig. 2C and Fig. 4G due to oversights during the figure preparation process.

Specifically, we mistakenly used the same β-actin WB band, resulting in the duplication of the two bands in Fig. 1E and Fig. 2C. Additionally, the WB bands for ERK1/2 and p-ERK1/2 were mistakenly placed, resulting in inadvertent duplication of the Ras WB band in Fig. 4G.

Amended Figure 2
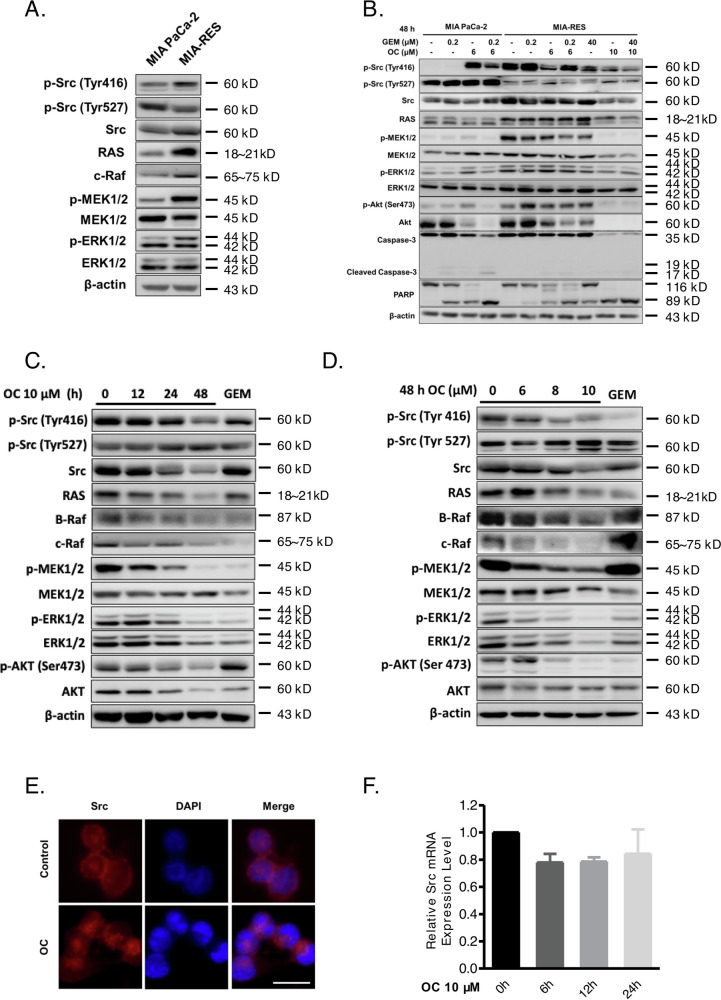


Original Figure 2
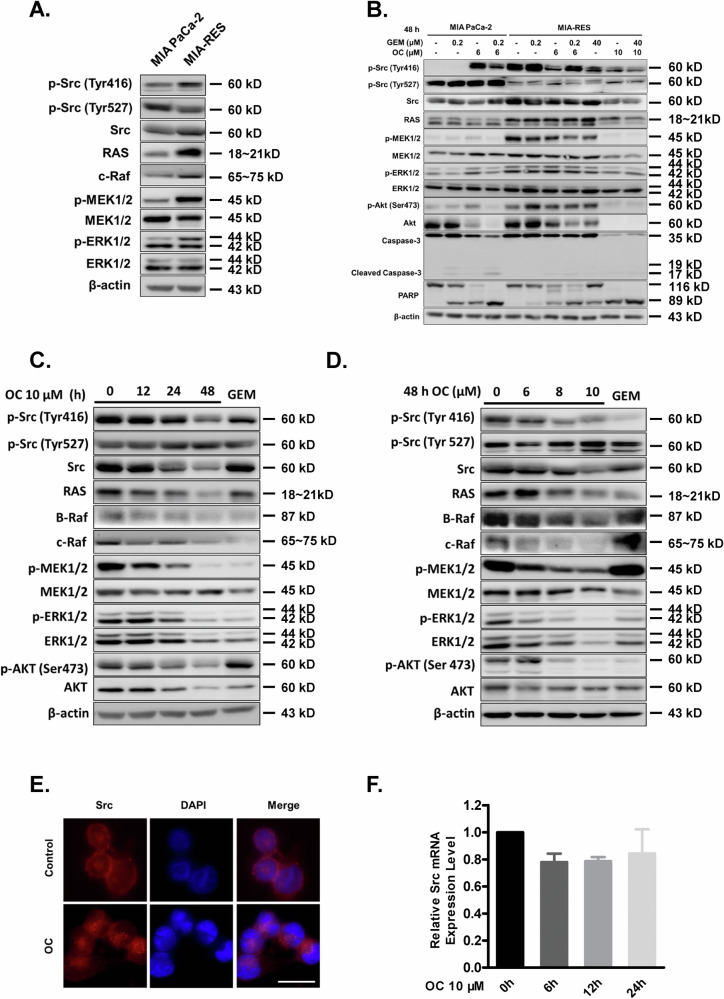


Amended Figure 4
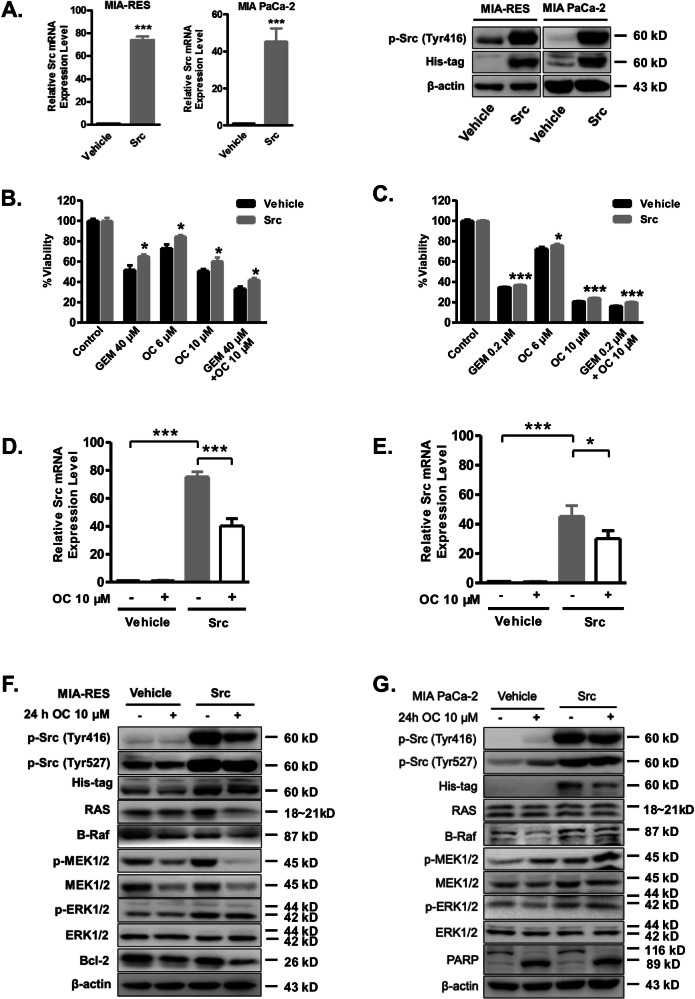


Original Figure 4