# Supplementary material for: Natural compound Oblongifolin C confers gemcitabine resistance in pancreatic cancer by downregulating Src/MAPK/ERK pathways
Source: Cell Death Dis. 2018 May 10;9(5):538. doi: 10.1038/s41419-018-0574-1 (PMC5970202; doi:10.1038/s41419-018-0574-1)
Supplement: Supplementary file 7 — Supplementary figure legends [file 41419_2018_574_MOESM7_ESM.docx]

**Supplementary figure and table legends**

**Supplementary Figure 1. OC inhibits the proliferation of parental and GEM-resistant PC by inducing G0/G1 arrest and apoptosis*.*** The cell viability of the parental human pancreatic cancer cell line Capan-1 and its GEM-resistant subline Capan-1-RES were exposed to different concentrations of GEM (A) and OC (B) for 48 h, which were determined by a CCK8 assay. Representative of flow cytometry MIA PaCa-2 (C) and MIA-RES cells (D), which were treated with indicated concentrations of OC for 24 and 48 h, and subjected to flow cytometry analysis using propidium iodide (PI) staining. (E) Indicative cell images were photographed by confocal microscopy using a × 40 magnification (Scale bar: 20 *μ*m).

**Supplementary Figure 2. Knockdown of Src plus OC increases chemosensitivity, whereas overexpression of Src reverts the effect of OC and enhances the chemoresistant of pancreatic cancer cells.** (A) Capan-1-RES and Capan-1 cells were transfected with non-targeting siRNA (NC) or Src-targeting siRNA (Src siRNA) for 48 h, and 2 μg Src plasmid or empty vector (Vehicle) for 24 h (B). The expression of Src mRNA (upper) and protein (down) were detected by qPCR and western blot analysis. TBP served as an endogenous control. *β*-actin was used as a loading control. Cell viability levels of Capan-1-RES (C) and Capan-1 cells (D) were measured by CCK8 assay in Src-silenced (Gray bars) and non-silenced cells (Black bars). Cell viability levels of Capan-1-RES (E) and Capan-1 (F) were measured by CCK8 assay in Src overexpression (Gray bars) and empty vector (Black bars). Treatments are indicated at the bottom of the graph for 48 h. Data represent the average of three independent experiments ± standard deviation. *p < 0.05 and **p < 0.01 ***p < 0.001

**Supplementary Figure 3. OC suppresses orthotopic human pancreatic tumor growth.** (A) Tumor tissue were removed from the mice which were orthotopically implanted with MIA PaCa-2 (Scale bar: 2 cm) and MIA-RES cells (Scale bar: 1 cm). (C,D) Statistical graphs of the immunohistochemical staining of Ki-67, Cleaved caspase-3, Src and p-Src (Tyr416) from the indicated. *P < 0.05, **P < 0.01, ***P < 0.001 compared to the control (n = 6). The percentages of IHC staining positive cells were calculated from five random fields.

**Supplementary Table 1. List of primary antibodies used in this study.** Abbreviations: CST: purchased from the Cell Signalling Technology, Danvers, MA, USA; SCB: Santa Cruz Biotechnology, Santa Cruz, CA, USA.

**Supplementary Table 2.** **The oligonucleotide sequences of control siRNA and c-Src siRNA (h), which composed by four duplexes of specific siRNA.**

**Supplementary Table 3. Oligonucleotide sequences of qRT-PCR primers.**
